# iPhy: an integrated phylogenetic workbench for supermatrix analyses

**DOI:** 10.1186/1471-2105-12-30

**Published:** 2011-01-24

**Authors:** Martin O Jones, Georgios D Koutsovoulos, Mark L Blaxter

**Affiliations:** 1 Institute of Evolutionary Biology, University of Edinburgh, Edinburgh EH9 3JT, UK

## Abstract

**Background:**

The increasing availability of molecular sequence data means that the accuracy of future phylogenetic studies is likely to by limited by systematic bias and taxon choice rather than by data. In order to take advantage of increasing datasets, user-friendly tools are required to facilitate phylogenetic analyses and to reduce duplication of dataset assembly efforts. Current phylogenetic pipelines are dependency-heavy and have significant technical barriers to use.

**Results:**

Here we present iPhy, a web application that lets non-technical users assemble, share and analyse DNA sequence datasets for multigene phylogenetic investigations. Built on a simple client-server architecture, iPhy eases the collection of gene sets for analysis, facilitates alignment and reliably generates phylogenetic analysis-ready data files. Phylogenetic trees generated in external programs can be imported and stored, and iPhy integrates with iTol to allow trees to be displayed with rich data annotation. The datasets collated in iPhy can be shared through the client interface. We show how systematic biases can be addressed by using explicit criteria when selecting sequences for analysis from a large dataset. A representative instance of iPhy can be accessed at iphy.bio.ed.ac.uk, but the toolkit can also be deployed on a local server for advanced users.

**Conclusions:**

iPhy provides an easy-to-use environment for the assembly, analysis and sharing of large phylogenetic datasets, while encouraging best practices in terms of phylogenetic analysis and taxon selection.

## Background

In recent years, phylogenetic studies involving large numbers of taxa and loci (and hence large numbers of characters) have proven able to resolve taxonomic uncertainties that were previously intractable [1-3]. For some large, well-studied groups (e.g. Nematoda [4]), the most comprehensive current taxonomies are based on a single locus (typically small subunit ribosomal DNA), but it is clear that single loci are unlikely to retain enough signal to resolve all relationships at all levels within a phylogeny containing large numbers of taxa [5,6]. For these analyses, additional loci must be added to the dataset. Even in analyses including many loci, major taxa of interest may be represented by single species, whose idiosyncratic evolutionary trajectories may strongly bias the resulting phylogenetic hypotheses and biological inferences made from them. Testing of the new phylogenies requires sampling of multiple species per taxon of interest, and many genes per taxon.

These supermatrix approaches to phylogenetic problems offer the promise of high resolution at all taxonomic levels, with details of recent and distant divergences provided by rapidly and slowly evolving molecular characters respectively. For many groups, no single locus has yet been sampled across all important taxa, therefore no single-gene phylogeny will be able to include all the relationships of interest. The incomplete nature of large multigene datasets, which must, by necessity, contain missing data, is now thought to be unproblematic under modern methods of phylogenetic reconstruction [7]. The extra information that an incompletely-sampled locus can contribute to the dataset outweighs the potential for added noise.

Phylogenetic reconstruction is vulnerable to being misled by systematic biases, sequence characteristics that are not accounted for by evolutionary models and that affect all characters from an organism's genome. Such biases are particularly problematic when carrying out large-scale phylogenetic reconstruction, as they are 'actively misleading': support for the incorrect relationships grows with increasing amounts of data. Such biases include between-species heterogeneity of evolutionary rates (where accelerated evolutionary rates lead to the phenomenon of long branch attraction [8]) and base and amino acid composition [9,10]. Due to the systematic nature of these biases, simply adding additional loci for phylogenetic reconstruction does not help to eliminate them. However, the large volume of public sequence data could be mined to avoid biased taxa by selecting the least-biased representatives of a taxonomic group for phylogenetic analysis. Such a strategy relies on first assembling a multigene dataset for all species in the taxa of interest, then selecting a set of species for phylogenetic analysis based on sequence characteristics known to be important for phylogenetic reconstruction.

Collation of these large, multigene datasets represents a significant investment of time and effort and once such a dataset is assembled, it represents a valuable resource for future work. As phylogenetic methods and models improve, the dataset could be re-analysed using new models of evolution and methods of phylogenetic reconstruction. Alternatively, researchers with an interest in a particular phylogenetic question may wish to analyse a subset of a larger dataset in more detail using methods that cannot feasibly be applied to the entire dataset. This is particularly true for several very large datasets recently described for Metazoa [2] and Arthropoda [1], both of which contain large amounts of sequence data for important groups that could be a fruitful target for more detailed investigation. However, the potential value of these datasets is currently not achieved, due mostly to the obstacles to sharing and reanalysing them. Typically, large datasets are assembled using *ad hoc *bioinformatics methods, stored inaccessibly and managed using informatics tools that require a high degree of technical ability. Some integrative tools have been published: for example, SCaFoS [11] takes individual gene alignments and either selects a single representative sequence or constructs a chimeric sequence for each group of interest. It then allows genes to be selected for inclusion in a final alignment based on their level of missing data. However, monophyletic groups (for which sequences can be combined) must be provided by the user along with multiple sequence alignments for each locus of interest.

Community endeavours such as TreeBase [12] offer a means to store both phylogenetic analyses and the underpinning data matrices, but are limited in their functionality, particularly in terms of searching by systematic group, and barriers to merging of complementary datasets. It is also possible to store multiple sequence alignments in EMBL/GenBank/DDBJ [13-15], and thus access the integrative data query tools of EMBL-EBI or NCBI, but the trees derived from analyses of these datasets are not stored. TreeFam [16] is a project that aims to build and maintain phylogenetic trees for all protein families defined by the Pfam [17] database. The alignments underpinning TreeFam phylogenies are thus available, but the site does not record phylogenies derived from supermatrices of multiple genes. iTOL (the interactive Tree Of Life [18]) is a web-based, client-server system that allows users to upload and store phylogenetic trees, and to use the sophisticated data visualisation and agglomeration tools available to produce striking, publication-ready graphics. While phylogenies and metadata can be shared on iTOL, underpinning alignment files are not, and it is not a workbench for assembling new supermatrices. New tools are required to better exploit the efforts and skills of phylogenetics researchers.

In light of these considerations, we have developed a user-friendly, client-server approach to collecting and sharing phylogenetic raw data (alignments) and products (trees). The tool collates a dataset by importing DNA sequences and assigning them to known loci. Our driving principles have been the production of an attractive and intuitive tool that can be used by researchers whatever their levels of bioinformatics skills and represents accepted best practices in the workflow with an open attitude to incorporation of alternative approaches. Our solution uses curated database metadata intelligently, efficiently stores both *a priori *and derived systematic and phylogenetic trees, and facilitates data sharing and presentation. Here we introduce this tool, iPhy (an interactive phylogenetic workbench), and demonstrate its use.

## Implementation

The iPhy workflow is carried out in two stages - dataset assembly, and subset analysis. In dataset assembly, the loci and taxonomic groups of interest are defined, DNA sequence data are added, and consensus sequences are built. Since iPhy does not address the problem of identifying orthologous sequences, this stage relies on existing annotation, either taken directly from GenBank, or derived by upstream processing using orthology identification tools [19,20]. The resulting dataset can be shared simply by distributing a URL. In subset analysis, subsets of loci and taxa ("slices") are selected using various criteria, downloaded as concatenated multiple sequence alignment files, and analysed using external phylogenetic tools. The trees resulting from phylogenetic analysis can be uploaded to iPhy, which then adds detailed annotation and submits the trees to iTol for viewing.

**Figure 1:**
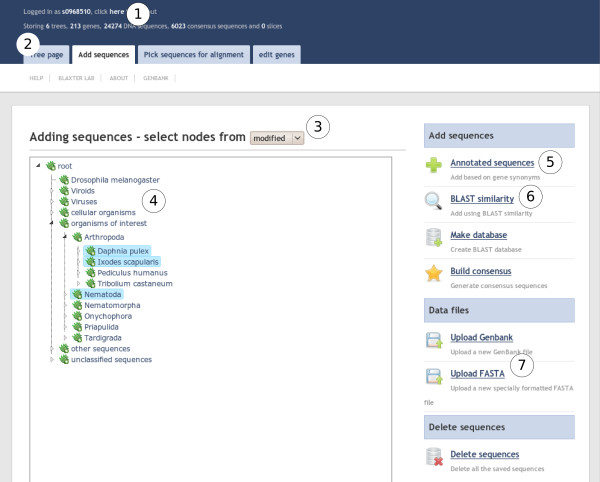
**The iPhy user interface**. The user is viewing the "Add Sequences" tab. The interface shows the current user name (1) and a set of summary statistics about the dataset (2). A modified taxonomy has been selected (3) which is displayed in the taxonomy panel (4). The user has expanded several nodes of the taxonomy and selected several nodes. Sequences belong to the selected nodes, or their descendants, will be added to the current dataset based on either annotation (5) or similarity to known sequences (6). From this panel the user can also upload new data files (7).

iPhy is implemented as a web application and is written in Groovy using the Grails web framework (Figure 1 shows part of the user interface). It uses the BioJava library for file parsing [21], the Gpars library for parallelization, and the ACEGI Grails plugin for user management. It is offered as a publicly-available server at iphy.bio.ed.ac.uk, and is also available to download for local installation.

### Defining loci

In order to assign sequences to loci (genes), iPhy requires a list of locus or gene names and their synonyms. iPhy contains a simple tool, suitable for manually curating a small list of loci. For larger numbers, including cases where the loci are mined from existing literature, gene names and synonyms can be collated manually or taken from online databases such as NCBI Homologene [13], and then uploaded in Comma Separated Value (CSV) format (see additional file 1: synonyms.csv).

### Defining taxa

iPhy is designed to be taxonomically aware and a taxonomic tree is used at several stages in the workflow. By default, iPhy uses the NCBI taxonomy [13], but users are free to define multiple taxonomic trees by modifying this core taxonomy. This allows iPhy to take advantage of the NCBI systematic taxonomy where the user judges it to be accurate (or irrelevant to the phylogenetic hypotheses under investigation) but to overrule it when desirable. The user can create, rename and move nodes (and their associated subtrees) in a drag-and-drop interface. Nodes in user-defined taxonomies need not correspond to traditional biological systematic groups, allowing operational taxa such as "organisms with a draft genome sequence" or "parasites" to be created, and, since multiple taxonomies can be defined for a single dataset, the user can organise species (and hence sequences) in whatever way is most helpful.

### Importing sequences

iPhy can import DNA sequence data from GenBank format files (including compressed GenBank release files downloaded from the NCBI web server). Protein-coding and RNA gene features corresponding to the list of known loci are extracted from the GenBank records and stored. Records that lack annotation (e.g. expressed sequence tags (ESTs) derived from sequencing of mRNAs), or that have annotations that do not match locus names or synonyms, can be identified and imported where relevant using BLAST [22]. iPhy can generate a BLAST database of annotated sequences (ones that have metadata matching the list of locus names and synonyms), and the unmarked sequences are searched against this. All sequences, whether identified by annotation or BLAST similarity, are screened to ensure that they belong to the selected set of taxa (which can be species or higher-level groups), allowing whole GenBank release files to be processed without adding large volumes of data for organisms not of interest. iPhy can also import DNA sequences from FASTA [23] format files where the sequence header follows a simple naming schema (see additional file 2 : sequences.fsa), allowing sequence data produced by third-party tools (such as assemblies of ESTs) to be imported.

### Building consensus sequences

Consensus sequences are generated whenever multiple sequences are available for a particular locus in a particular species. There are two exceptions to this rule: if one of the annotated sequences is derived from a fully sequenced genome (as determined from the 'description' field of the GenBank record), it will be used for the consensus and all others will be ignored. Similarly, a single annotated sequence takes precedence over a collection of sequences identified on the basis of BLAST similarity. For derivation of the consensuses, CAP3 [24] is used with default settings to assemble all available sequences into a contig. In the event that multiple contigs are produced (e.g. in the case where a sequence in GenBank is incorrectly annotated), the longest contig is selected as the consensus.

### Data review and summary

iPhy allows the user to review the distribution of sequences per species (or higher group) and locus to assist in checking the sanity of the collated dataset, and to streamline subsequent analyses.

### Picking subsets for analysis

Once a dataset has been assembled, subsets of species and loci ("slices") can be selected for analysis. Multiple slices can be defined for each dataset, and these are stored within iPhy. Within iPhy, the user can manually select a set of taxa (using any of the previously-defined taxonomic trees) and loci. Each species that is chosen is included in the slice, and where a higher-level taxonomic group is chosen, all descendent species are included in the slice. Alternatively, the user can let iPhy select representative taxa from different taxonomic groups for inclusion in the slice based on explicit criteria. In this scenario, the user selects (1) a set of nodes from one of the previously-defined taxonomic trees, (2) the taxonomic level of interest [order, family, etc], (3) the number of representative species to be included per group at this level, (4) a list of loci and (5) the selection criterion. iPhy will recursively traverse the taxonomy, retrieving all nodes of the chosen level ('level nodes') that are descended from the selected nodes. For each level node, a list of candidate representative species is obtained from the taxonomy, and the candidates ranked according to the inclusion criterion. Finally, the chosen number of highest-ranking candidates are added to the slice. Currently, iPhy implements three selection criteria, designed to favour species that have desirable characteristics for deep phylogenetic reconstruction: number of characters, base composition, and evolutionary rate.

#### Number of characters

Under this criterion, candidate species are ranked according to the total number of characters present in the sequences across all selected loci for that species. The presence or absence of individual loci are not taken into account, so it is possible for this criterion to choose a candidate that has fewer loci but more characters than a rival (as in the case where the rival species have partial sequences).

#### Base composition

This criterion involves a preprocessing step, where the mean AT content is calculated across the entire dataset for each selected locus separately and stored as the 'global mean AT content'. To calculate the score for a candidate, the absolute difference between its AT content and the global mean is calculated separately for each locus. The mean of these differences is the score: candidates with lower scores are ranked more highly. This criterion favours species where the AT content is most similar to that of the dataset as a whole. Only candidates with at least one sequence present are considered.

#### Evolutionary rate

For this criterion, a rapid multiple sequence alignment is carried out for each selected locus using MUSCLE [25] with the "*- maxiters 1*" parameter. To calculate the score for a candidate species, each locus is processed separately. A simple similarity metric is calculated between the aligned sequence for the candidate and the aligned sequences of ten other species, randomly selected from outside the level node. The similarity score is measured as the proportion of candidate sequence sites that are exact matches with the other sequence. The mean of these ten scores is the candidate's score for this locus. The mean of these scores over all loci is the candidate's overall score. Candidates with higher scores (i.e. higher average similarity to other species in the dataset) are ranked more highly.

### Alignments

Once a slice has been defined, the sequences for each locus are aligned using MUSCLE with the default parameters. To optimize alignment accuracy, a multi-step protocol is used. First, all sequences flagged as full-length in the GenBank records are translated. These protein translations are aligned, and the protein alignment is used to back-align the original DNA sequences using Tranalign from EMBOSS [26]. Second, all remaining partial DNA sequences are profile-aligned to the first-stage alignment. If no full length sequences are available for a given locus in the slice, the first step is omitted and all sequences are simultaneously aligned at the DNA level. Alignments can be viewed and edited for each locus separately using an embedded JalView [27] applet.

### Export for phylogenetic analysis and import of analysis results

iPhy does not perform phylogenetic analysis, as this process is well served by a wide range of algorithms and efficient software tools [28-30]. Iphy assists the user in using these external tools by producing analysis-ready data files and importing the post-analysis output files.

The aligned sequence supermatrix for a slice is first generated and downloaded. Individual locus alignments are concatenated and written to a NEXUS [31] format file. The NEXUS format allows the alignment to be divided up into partitions, and iPhy specifies partitions for each locus individually and, where possible, for each codon position within each locus. The user then analyses this supermatrix with their method of choice, ensuring that the analyses are saved as the standard output formats for each program.

### Tree annotation

By default, iPhy names the supermatrix NEXUS file using the internally stored identifier for the slice. Most phylogenetic reconstruction tools generate output tree files with a similar name to the alignment file, and thus a file containing a tree derived from a slice supermatrix can be easily identified. iPhy can export these saved trees for visualisation in iTol. As iPhy uses the NCBI taxon identifiers (or "txid") as the leaf node names, this allows iTol to automatically assign scientific names to the leaf and internal nodes of the tree. iTol is a rich environment for tree annotation, and iPhy takes advantage of this to decorate uploaded trees with metadata. Currently, iPhy offers decorations of exported trees showing the number of characters for each locus for each species as a stacked bar chart, and the AT content for each locus as a heat map. Tree files in Newick, Nexus and PhyloXML [32] format can be annotated and uploaded to iTol.

## Results

### Assembly of a large nematode dataset

As mentioned above, the current molecular phylogenetic hypothesis for Nematoda is based almost entirely on a single locus, the small subunit ribosomal RNA (ssu rRNA) gene [4], and it is likely that the utility of this marker has been saturated. To assemble a larger dataset for nematodes, a list of locus names was mined from two recent multigene phylogeny publications [1,2]. For each locus, the NCBI Homologene database [33] was used to look up the Homologene ID and to obtain a list of synonyms. The list was then manually curated to ensure that the maximum number of correct synonyms were included for each locus. We removed synonyms that appeared in multiple loci or were known to be incorrect and added known synonyms that were missing. Compressed GenBank format files for the *gb-inv *(invertebrate) division of GenBank were downloaded from the NCBI FTP server and processed using iPhy to extract sequences of interest from Nematoda, *Drosophila melanogaster*, and other potential ecdysozoan outgroup taxa (Onychophora, Tardigrada, Priapulida, and Nematomorpha). These annotated sequences were also used to construct a BLAST [22] database containing protein sequences. The redundancy of the BLAST database was reduced using cd-hit [34]. EST data were retrieved for 60 nematode species from NEMBASE4 [35], and supplemented with unpublished data for five additional species (nematodes *Anguillicoloides crassus *and *Laxus oneistus*, and ecdysozoa *Euperipatoides kanagrensis, Gordius robustus, Priapulus caudatus *and *Gordius aquaticus*).

**Figure 2:**
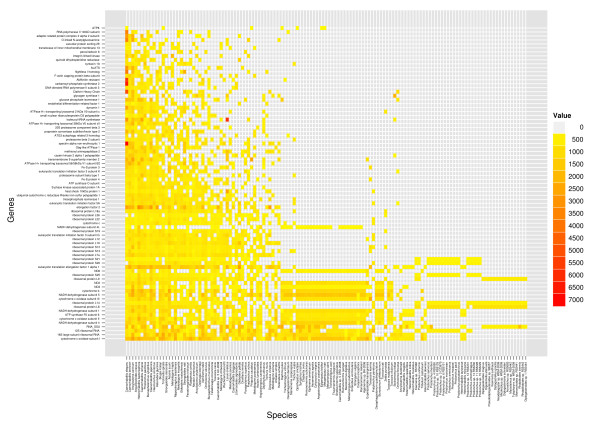
**A supermatrix for Nematoda assembled in iPhy**. The colour of the box at each intersection shows the length of the consensus DNA sequence for a given gene in a given species. Genes and species are ordered by total number of characters. A high-resolution version of this figure is available as additional file 4 : heatmap.svg.

These data were screened for sequences of interest using the prepared BLAST database. For sequences retrieved from NEMBASE4, protein translations were available and the BLASTP algorithm was used; for other sequences, BLASTX was used. As we expect different loci to evolve at different rates, the cutoff bitscore for gene identification was empirically determined for each locus individually. We used the bitscore of the match between the annotated genes from *Caenorhabditis elegans *and *D. melanogaster *as the cutoff bitscore for each locus, on the basis that sequences within Nematoda will show greater similarity to *C. elegans *than the *D. melanogaster *sequence did. In total, 24528 sequences were gathered for 81 loci. Consensus-building by iPhy resulted in a final dataset of 8326 consensus sequences with the expected patchy distribution across taxa (Figure 2).

### Subset selection and analysis

To generate subsets of the data for phylogenetic analysis, the three most highly-represented protein coding genes were selected (ribosomal protein L8 (rpL8), rpL9 and rpL14). iPhy was used to build subsets including these three genes and one representative species from each nematode order as defined by the NCBI taxonomy, along with *D. melanogaster *as an outgroup. *Trichinella spiralis *was excluded from these subsets as its extremely high AT content and accelerated evolutionary rate obscured the results in preliminary investigations (not shown). Slices were generated using each of the three criteria implemented in iPhy (referred to hereafter as *most_chars*, *least_bias *and *slowest_rate*). For each subset, phylogenetic analysis was carried out using MrBayes 3.1 [29] using a GTR+G+I model with separate model parameters for each gene partition and two independent runs of 4 chains for 500000 generations. The runs were checked for convergence and stationarity before summarizing trees with the first 50000 generations discarded as burn in. To investigate the effects of the different selection criteria, the standard deviation of AT content was calculated for each gene for each subset, and for the dataset as a whole. Maximum Likelihood estimates of tree length were obtained from the model parameter summaries produced by MrBayes (Table 1).

**Table 1:** Descriptive statistics of subset alignments and trees

	L14 AT content		L8 AT content		L9 AT content		Tree length	
	*Mean*	*S.D*.	*Mean*	*S.D*.	*Mean*	*S.D*.	*Mean*	*Variance*
whole dataset	0.5118	0.0819	0.4686	0.0675	0.5221	0.0745		

*most_chars*	0.5614	0.0750	0.5175	0.0740	0.5593	0.0600	3.837	0.0500
*least_bias*	0.5266	0.0570	0.4845	0.0524	0.5206	0.0461	3.489	0.0374
*slowest_rate*	0.5182	0.0692	0.4860	0.0678	0.5334	0.0503	3.033	0.0375

Each row shows a set of statistics associated with a given subset of the data, or with the entire dataset. Each row shows the mean and Standard Deviation of AT content for each gene separately, and the mean and variance of the total tree length as reported by MrBayes.

**Figure 3:**
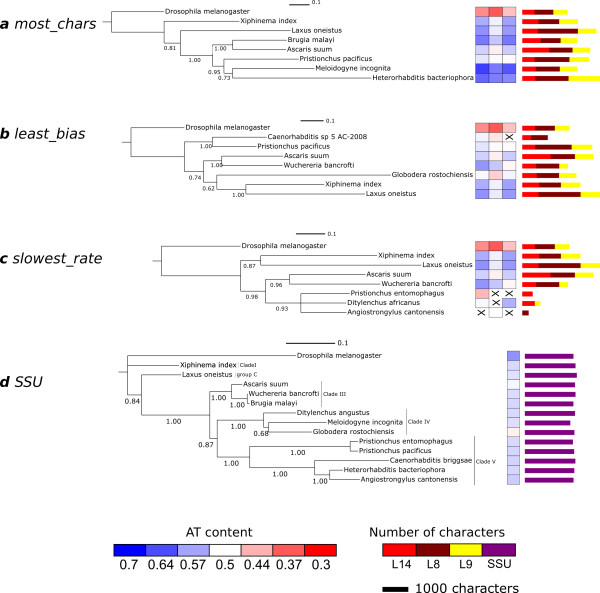
**Analyses of slices from the Nematoda dataset**. The figure shows the results of analyses of automatically selected taxon subsets from the Nematoda dataset using various criteria for a three-gene supermatrix. (A) *most_chars *species (one per order) with most characters for the three genes; (B) *least_bias *species showing the lowest base composition bias; (C) *slowest_rate *species with the inferred slowest overall rate of evolution. (D) For comparison, we show the tree derived from alignment of full length SSU rRNA sequences for twelve of the fourteen species included in the iPhy slices in parts (A), (B) and (C). For two of the species (*Caenorhabditis sp. 5 *and *Ditylenchus africanus*) no SSU rRNA sequence was available so we have included closely-related species (*Caenorhabditis briggsae *and *Ditylenchus angustus*). Clade membership *sensu *Blaxter 1998 [36] is shown on the tree. For each iPhy subset the figure shows, from left to right, the tree resulting from phylogenetic analysis; a heat map showing the AT content of each of the three genes; a stacked bar chart showing the number of characters for each gene. Scale bars above each tree show the branch length associated with 0.1 changes per site. Order names are given in parentheses. The keys at the bottom of the figure show, from left to right, the mapping of colours to AT content for the heatmap, and the mapping of colours to loci for the bar chart. The scale bar shows the length of bar representing 1000 characters.

The subset where species were selected on the basis of the number of characters available (Figure 3A) contained the most characters, and was the only subset where every gene was present for every species. This subset had the highest standard deviation in AT content among species for all three loci, and produced the tree with the greatest overall length. The subsets where species were selected for low AT content bias (Figure 3B) and low evolutionary rate (Figure 3C) showed the expected AT content and tree length characteristics.

The most_chars tree (Figure 3A) recapitulates the tree derived from ssu rRNA (Figure 3D) and supported by morphological analyses, except that the tylenchid *Meloidogyne incognita* (in Clade IV of Blaxter et al. [36]) is sister (albeit with low posterior probability support) to *Heterorhabditis bacteriophora* (Clade V) and nested within Clade V taxa. *M. incognita* is known to have AT-bias issues. The least_bias slice (Figure 3B) resulted in a poorly-resolved tree, with two of five internal nodes with posterior probabilities less than 0.75. While relationships between Clade V (*Caenorhabditis elegans* and *Pristionchus pacificus*) and Clade III (*Ascaris suum* and *Wuchereria bancrofti*) taxa were strongly supported, the placement of Clade IV (*Globodera rostochiensis*), group C (*Laxus oneistus*) and Clade I (*Xiphinema index*) species is in conflict with morphological and ssu rRNA data. The slowest_rate tree (Figure 3C) links Clade IV (*Ditylenchus africanus*) with Clade V (*Angiostrongylus cantonensis* and *Pristionchus entomophagus*), affirms Clade III and marginally supports group C being sister to Clade I. While these analyses are inconclusive, they show that new loci and new taxa can be added to analyses of large phylogenetic problems within iPhy, and multiple slices can be easily exported, analysed and visualised. We are now carrying out extensive multigene analyses of Nematoda using iPhy.

## Discussion

iPhy is designed from the ground up to facilitate data gathering and sharing, a task that we anticipate will become increasingly important due to recent and anticipated increases in the rate of sequence data generation. Sharing an iPhy dataset, along with its associated slices, is simply a matter of distributing a URL, in contrast to existing dataset assembly tools [11,37], which have multiple dependencies and complex, non-graphical interfaces. At the same time, the design of iPhy ensures that advanced users can integrate it with local pipelines. For example, more experienced users may want to assign sequences to loci using in-house tools (e.g. transcriptome assembly software [38] or existing orthology assignment software [19,20]). Where additional computing power is required, iPhy can be installed on a local server, making it easy to provide a dedicated installation for members of a research group. An administrator of a local installation can have direct access to the iPhy database and web application folders. This allows files to be directly transferred to the server (instead of being uploaded through a web browser), and queries to be run directly on the database (see additional file 3 : heatmap.pl for an example).

Understanding of the importance of systematic bias as a factor limiting the accuracy of phylogenetic studies seems likely to increase, as does the development of more sophisticated models to overcome biases. The development of explicit criteria for taxon and locus selection, as implemented in iPhy and elsewhere [11] is a promising step, though the criteria themselves could still be improved. In particular, when considering the degree of compositional bias, it would be beneficial to take into account the degree of variation around the global mean for a given locus, and assign more weight to loci where the overall dataset variation is low. Another potential improvement would be the ability to select sequences based on amino acid criteria. Additionally, the question of how best to deal with missing data in criterion selection remains open. It is likely that a composite measure, taking into account number of informative characters, compositional bias, and evolutionary rate, would perform better than any single criterion. Parallel developments for selecting informative regions from large alignments [39] also promise to assist in selecting the best subsets of large datasets for analysis, particularly where such subsets are generated in a largely automated fashion, as in iPhy.

The results of the Nematoda dataset analysis illustrate the trade-off that must be made when selecting species for phylogenetic analysis. For accurate phylogenetic reconstruction, we desire an alignment with a large number of characters, with homogeneous base composition, and with an absence of long branches. Optimising our taxon choice for any one of these three desiderata requires sacrificing the other two. Obviously, the usefulness of selection criteria for any given high level taxonomic goup is dependent on both the number of potential representative species and the degree of variation among them for the trait in question. Hopefully future sequencing efforts will fill in the taxonomic gaps to increase both of these factors, increasing the usefulness of selection criteria for increasing phylogenetic accuracy. Our results for Nematoda also indicate a degree of interaction between the three criteria. Favouring species with low AT content bias also favours slow evolutionary rates, and vice versa. This is not surprising, as we expect lineages that have undergone selection for extreme compositional bias to have long branches.

A significant problem for large dataset assembly, highlighted by our sample dataset, is the identification of synonyms and the reliability of annotation. While some databases of gene name synonyms exist, prospects for automatic synonym determination are bleak, and manual expert curation remains the gold standard. Additionally, annotation extracted from GenBank format files is not always guaranteed to be correct. The development of methods for automatic orthology assignment that do not depend on prior annotation is a promising approach in this field [19,20], particularly in light of next-generation sequencing methods that generate large quantities of un-annotated sequence.

### Outlook and future prospects

This first release of iPhy represents a new stage in the collation and publication of large-scale phylogenomic datasets. With the framework in place we intend to further develop this tool to integrate additional external resources, such as protein family [16] and orthologue definition databases [20], and additional output formats, such as ready-to-submit data files including trees and metadata aimed for inclusion in TreeBase [12] or other phylogenetic resources. In addition, by defining an API for the workbench, we will facilitate programmatic access to stored datasets, and drive the realisation of an continuously-revised phylogenetic engine, where new data are automatically added to old datasets and used for reanalysis and re-resolution of long-standing issues in evolution. Another possible useful extension to iPhy's feature set would be the ability to deal with amino acid sequence data.

## Conclusions

iPhy is the most accessible and user-friendly tool for phylogenetic dataset collation, analysis and sharing currently available. By making iPhy available as a web application, we significantly lower the barrier to its use, ensuring that anyone with a web browser can take advantage of this tool. By lowering the barriers to use, iPhy puts a powerful bioinformatics tool in the hands of researchers who would not otherwise have access to such software, while promoting best practices in terms of phylogenomic analysis, and making datasets available for reuse and reanalysis.

## Availability and requirements

Project name: iPhy

Project home page: iphy.bio.ed.ac.uk

Operating system: platform independent

Programming language: Groovy (with Grails web framework)

Other requirements for public web version: none

Other requirements for locally-installed version:

Java servlet container (e.g. Tomcat) or Grails 1.2

Third-party tools: BLAST, CAP3, Muscle

Java & Groovy libraries: BioJava, GPars, HTTP Client, json-lib, postgresql jdbc driver PotsgreSQL RDBMS

License: GNU GPL

Any restrictions to use by non-academics: no

## Authors' contributions

The project was conceived by MJ and MB. The software was designed and implemented by MJ. The Nematoda analyses were performed by GK. All authors read and approved the final manuscript. The authors declare no competing interests.

## Supplementary Material

Additional File 1 **List of gene synonyms**. An example file showing the format for uploading lists of gene synonyms to iPhy. This file contains the gene names and synonyms used to assemble the nematode dataset described in the manuscript. Click here for file

Additional File 2 **Input DNA sequences in FASTA format**. An example file showing the format for uploading DNA sequences to iPhy. The FASTA header for each sequence contains the locus name and organism taxid, allowing the sequences to be correctly assigned to locus and species. Click here for file

Additional File 3 **Perl script to draw a heat map showing sequence distribution**. A Perl script that queries the iPhy database to construct a heat map showing the distribution of sequence data across loci and taxa. This script was used to generate Figure 2 in the manuscript. Click here for file

Additional File 4 **Vector version of Figure 2**. A version of the heat map shown in Figure 2 in vector graphics format. This image can be zoomed without loss of resolution, allowing the heat map to be viewed at a large scale. Click here for file
